# 2020 JBSR Report

**DOI:** 10.5334/jbsr.2697

**Published:** 2021-11-25

**Authors:** Alain Nchimi

**Affiliations:** 1 Hôpital Universitaire Des Enfants Reine Fabiola (HUDERF), BE

**Keywords:** JBSR, BSR, Report, Impact Factor

## A new editor-in-chief arrives Jan 1, 2022

**Figure 1:**
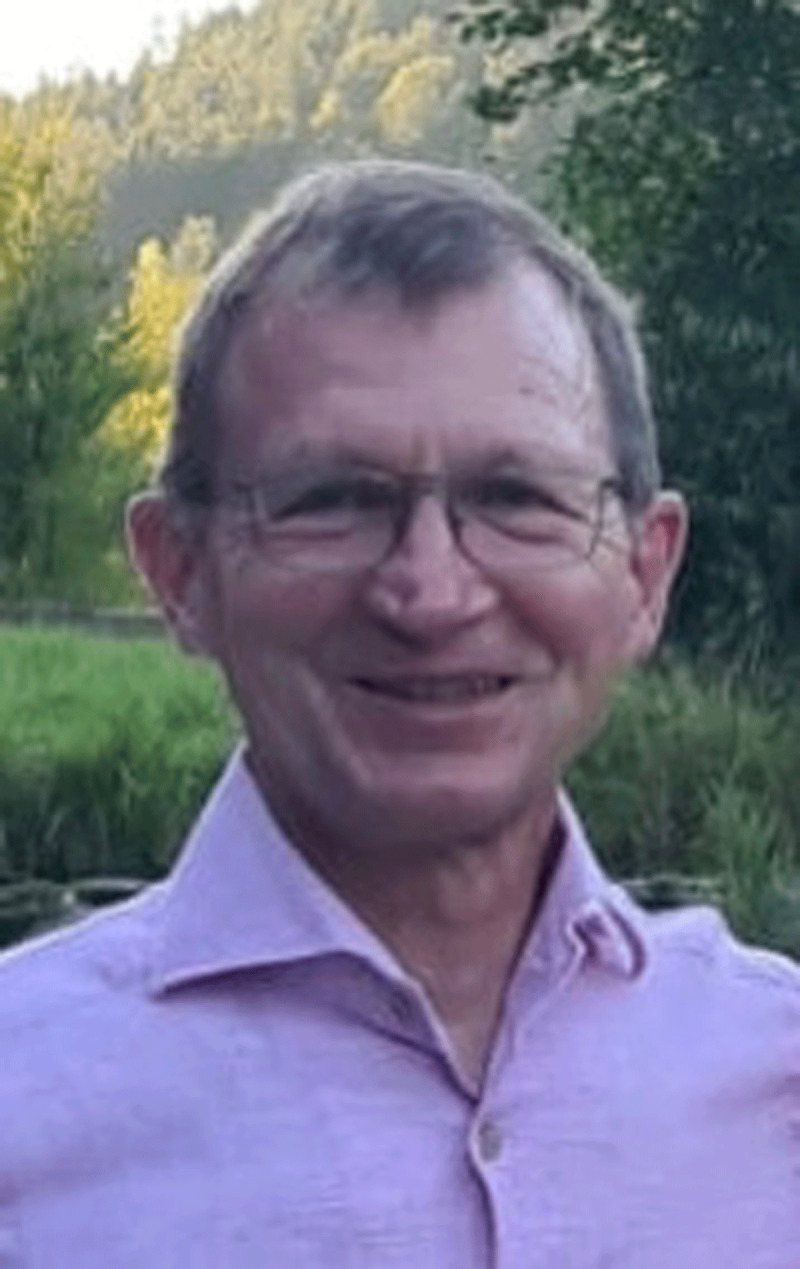
Prof. Raymond Oyen.

Prof. R. Oyen (***Figure 1***) has been selected by the board of the Belgian Society of Radiology (BSR) to take over as the new editor-in-chief of the *Journal of the Belgian Society of Radiology* (JBSR) Jan 1, 2022. R. Oyen, at 65 years old, graduated as a Doctor in Medicine in 1981, then as a specialist in radiology at the Catholic University of Leuven five years later. He was appointed full professor of radiology by the Catholic University of Leuven in 1994 and eventually chaired the clinical Department of Radiology of the University Hospital of Leuven for more than a decade, until September 2021. Since 1994, when de defended his PhD thesis entitled “Diagnosis and staging of prostatic carcinoma,” urogenital imaging has been the mainstay of an outstanding career in terms of research and teaching contributions. Professor R. Oyen is a co-founder and past president of the European Society of Urogenital Radiology (ESUR) and honorary member of the European Society of Gastrointestinal and Abdominal Radiology (ESGAR), the American Society of Abdominal Radiology (SAR), and the Asian Society of Abdominal Radiology (ASAR). Lastly, he is author or co-author of more than 300 publications and serves as reviewer for several prestigious journals.

## Turnaround times, the main challenge

As I wish a warm welcome to the new editor-in-chief, I believe there are no better hands than those of Prof. R Oyen to handle the job I’ll be leaving. My thoughts less relate to his fantastic career achievements and leadership than to the fact Prof. R. Oyen is already a highly committed associate editor of the journal, whilst having been for decades a prominent figure of the BSR and the current chair of its scientific council.

Holding these positions, he knows better than anyone that the challenge he’ll be facing as the new editor-in-chief of the JBSR lies in obtaining a stronger commitment of the scientific sub-specialties section chairs of the BSR to expedite the review process. Among all performance indicators in 2020 (one-year trend [1]), article turnaround times remain, respectively, 95 (+8%) and 65 (+18%) days for submission to acceptance or to rejection, and 25 (–232%) days for acceptance to publication (see the additional file for full report summary). Obviously, our editorial decision turnaround times could be cut further down, as only 32 out of 70 review requests were fulfilled on due time. The editorial decision delay issue is not specific to the JBSR as virtually all medical science journals editors owe struggles with peer-reviewing processes to several factors, including overcommitment to clinical duties and the overlooked value of peer-reviewing in academic curricula. The good news thus for the JBSR is that there is room for improvement under the leadership of the new editor-in-chief, especially in terms of finding incentives for the BSR scientific subspecialties section chairs to expedite review process.

## A growing audience among journals in radiology

The JBSR article content, length, and guidelines have now been unchanged for more than two years, offering a platform to sustain and even increase the trends that characterized the last five years, namely: Increasing pressure on manuscript quality, improving content originality and priority, and assisting young authors’ first publications.

In 2020, the JBSR received 374(+22%) submissions from all around the world and 337 submissions reached editorial final status. ***Table 1*** reports the decision rate per article category in 2020 as compared to the previous two years, which shows a steady decrease of the mean acceptance rate as a result of a higher selection that hits all categories, although unequally. The acceptance rate was around 10% for case reports and original articles, for which the main selling point is priority, whilst up to 40% of manuscripts valuable for image quality and teaching value (but less citation and originality), such as images in clinical radiology manuscripts, were accepted. The other reason images in clinical radiology manuscripts are still being considered for publication in the JBSR lies in the assumed policy of encouraging and assisting national young authors’ first publications.

**Table 1:** Acceptance and rejection rates for the JBSR (2018–2020) (N (%)).


YEAR	ACCEPTED	REJECTED	DESK REJECTED

2020	73 (21.7%)	124 (36.8%)	140 (41.5%)

2019	65 (23.3%)	101 (36.2%)	113 (40.5%)

2018	83 (34.6%)	31 (12.9%)	126 (52.5%)


**Figure 2:**
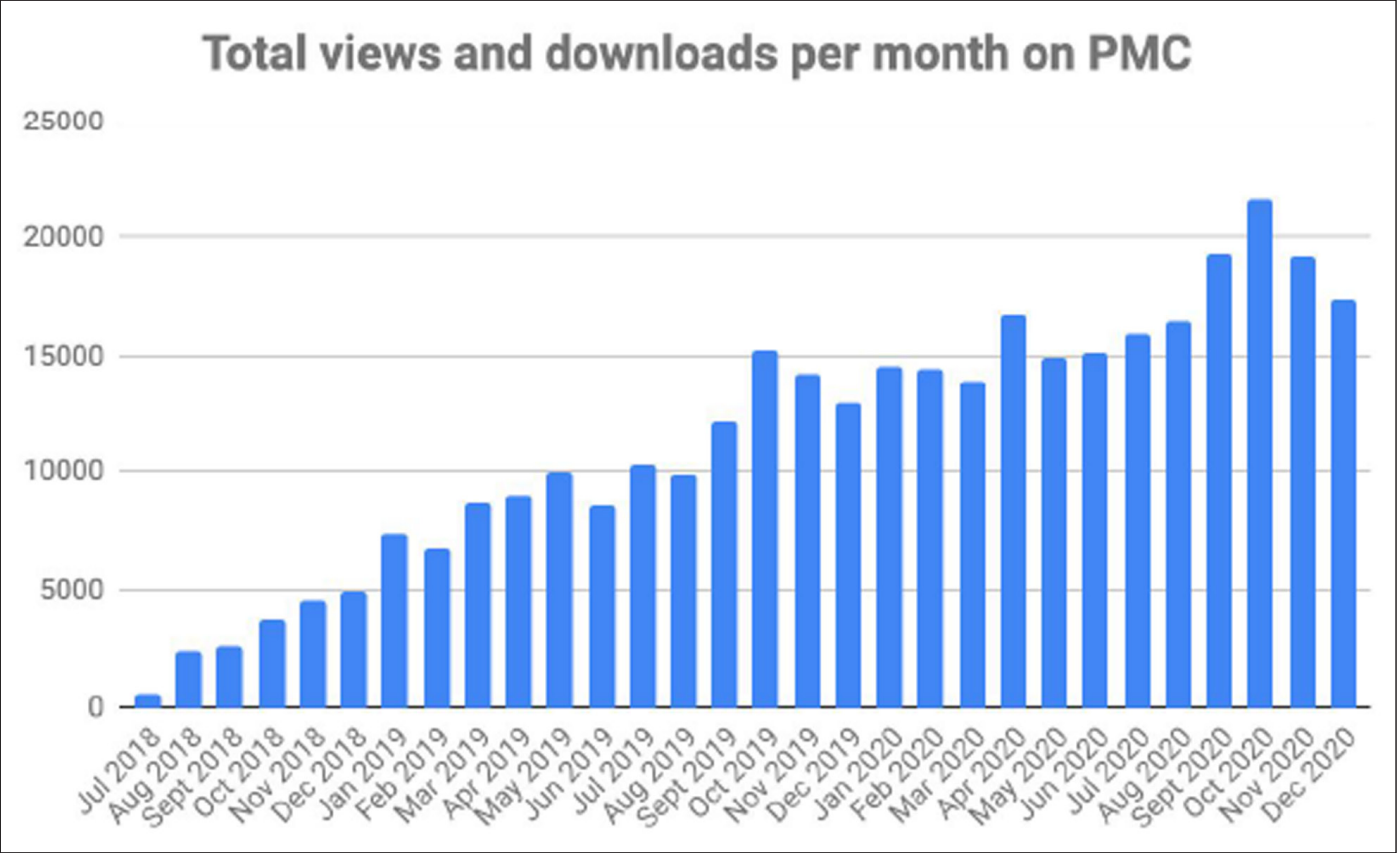
Combined JBSR article views and downloads on Pubmed Central per month in 2018–2020.

The selection pressure placed on submission probably resulted in a higher article quality that translated into a higher citation rate, with the journal impact factor reaching 0.729 (+53%) and then 1.894 (+160%) respectively in 2019 and 2020, ranking 101/134 in the Radiology, Nuclear Medicine & Medical Imaging category. The open access availability of the journal also probably accounts for such a progress rate. Indeed, the growth of article views and downloads both on the journal site and Pubmed Central (***Figure 2***) was sustained in 2020, again with a peak at the end of the year, around the date of the annual meeting of the Belgian Society of Radiology.
